# Supplementary material for: Hippo signaling differentially regulates distal progenitor subpopulations and their transitional states to construct the mammalian lungs
Source: bioRxiv. 2025 Oct 28:2025.10.28.684989. Preprint. [Version 1] doi: 10.1101/2025.10.28.684989 (PMC12636293; doi:10.1101/2025.10.28.684989)

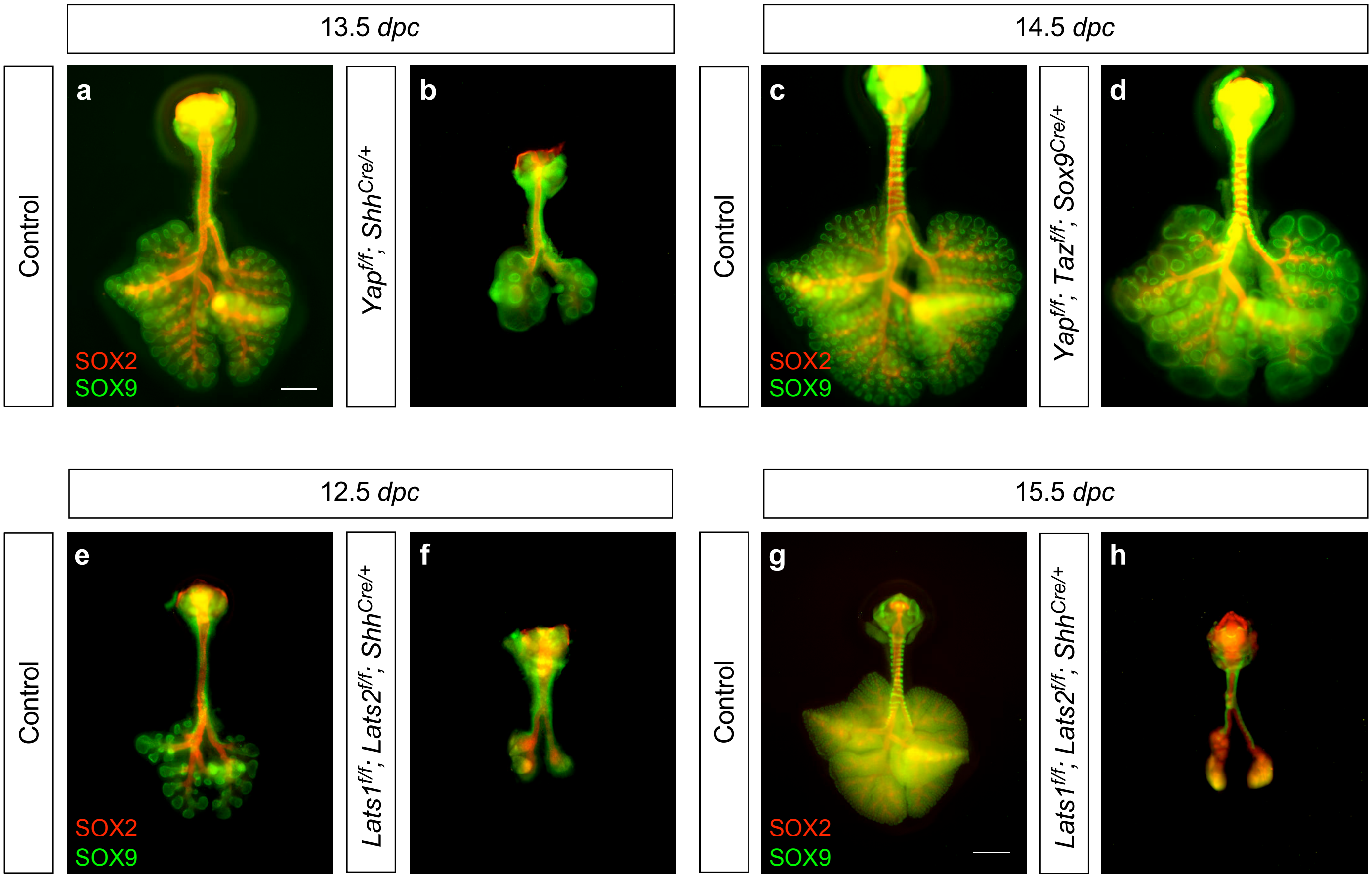

13.5 dpc
14.5 dpc
Control
a
Yap f/f; Shh Cre/+
b
Control
c
Yap f/f; Taz f/f; Sox9 Cre/+
d
SOX2
SOX9
12.5 dpc
15.5 dpc
Control
e
Lats1 f/f; Lats2 f/f; Shh Cre/+
f
Control
g
Lats1 f/f; Lats2 f/f; Shh Cre/+
h

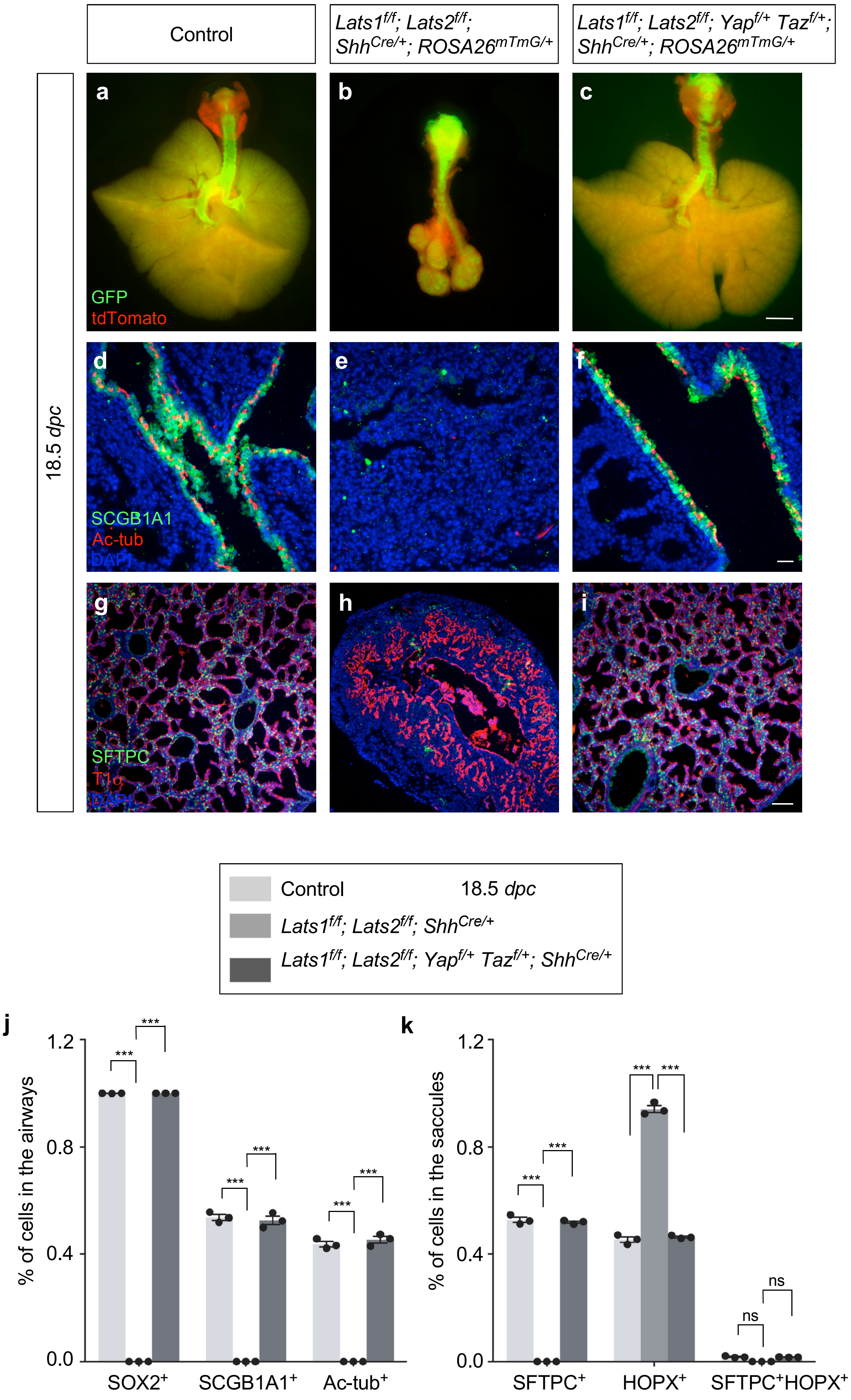

Control
Lats1f/f; Lats2f/f; ShhCre/+; ROSA26mTmG/+
Lats1f/f; Lats2f/f; Yapf/+ Tazf/+; ShhCre/+; ROSA26mTmG/+
18.5 dpc
a
b
c
GFP
tdTomato
d
e
f
SCGB1A1
Ac-tub
DAPI
g
h
i
SFTPC
T1α
DAPI
Control
18.5 dpc
Lats1f/f; Lats2f/f; ShhCre/+
Lats1f/f; Lats2f/f; Yapf/+ Tazf/+; ShhCre/+
j
k
% of cells in the airways
% of cells in the saccules
1.2
0.8
0.4
0.0
***
ns
SOX2+
SCGB1A1+
Ac-tub+
SFTPC+
HOPX+
SFTPC+HOPX+

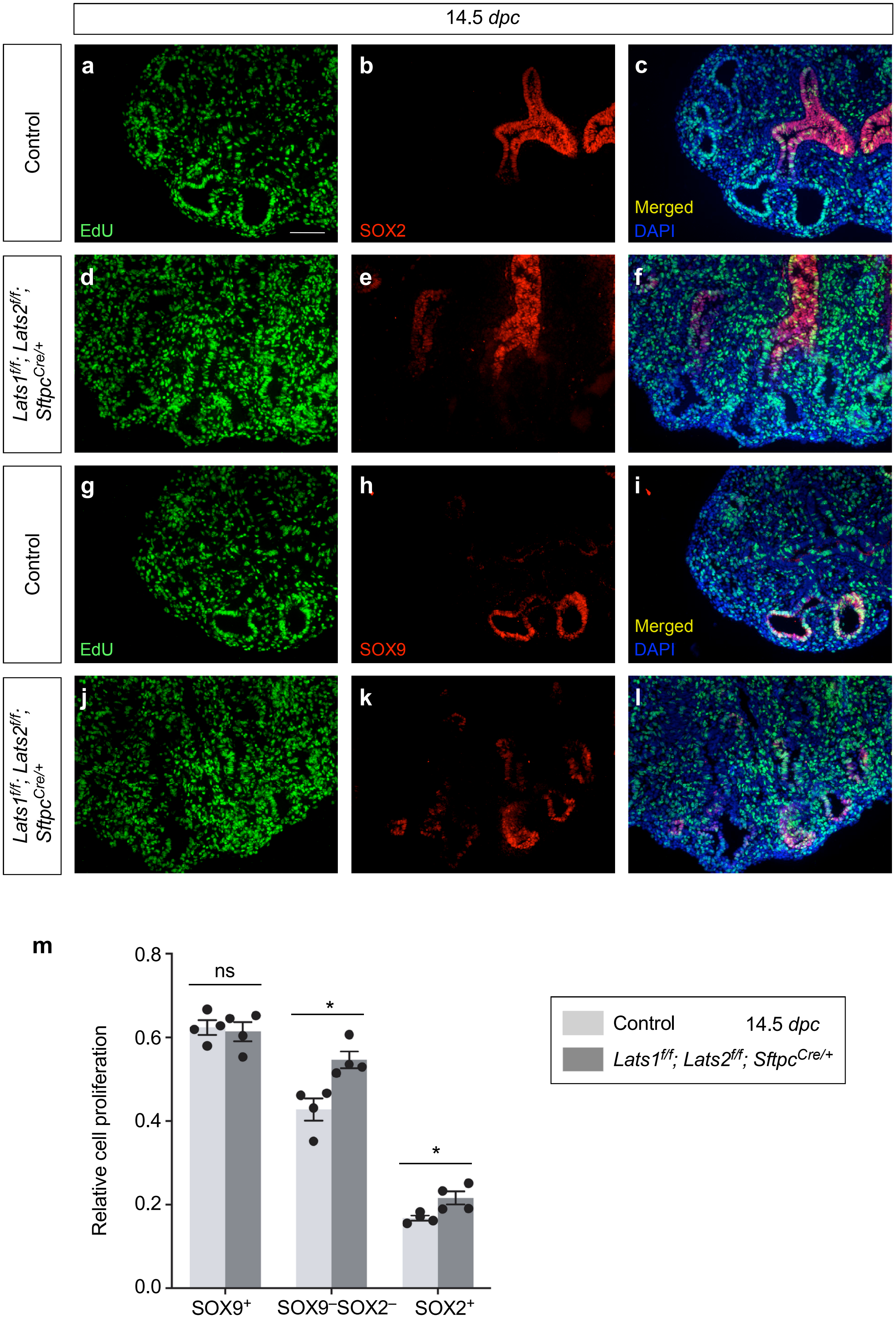
14.5 dpc
Control
Lats1f/f; Lats2f/f; SftpcCre/+
Control
Lats1f/f; Lats2f/f; SftpcCre/+
a
b
c
EdU
SOX2
Merged
DAPI
d
e
f
g
h
i
EdU
SOX9
Merged
DAPI
j
k
l
m
Relative cell proliferation
0.8
0.6
0.4
0.2
0.0
ns
*
*
SOX9+
SOX9−SOX2−
SOX2+
Control
14.5 dpc
Lats1f/f; Lats2f/f; SftpcCre/+

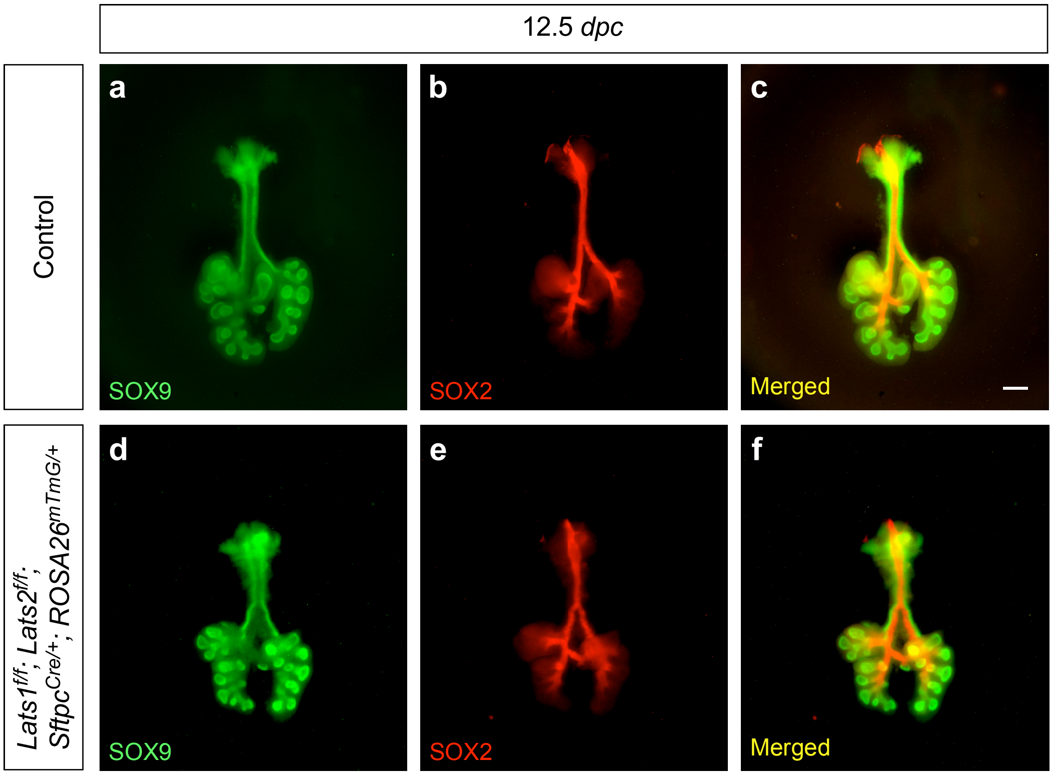
12.5 dpc
Control
Lats1f/f; Lats2f/f; SftpcCre/+; ROSA26mTmG/+
a
b
c
d
e
f
SOX9
SOX2
Merged
SOX9
SOX2
Merged

Zhang et al. Supplementary Figure 5

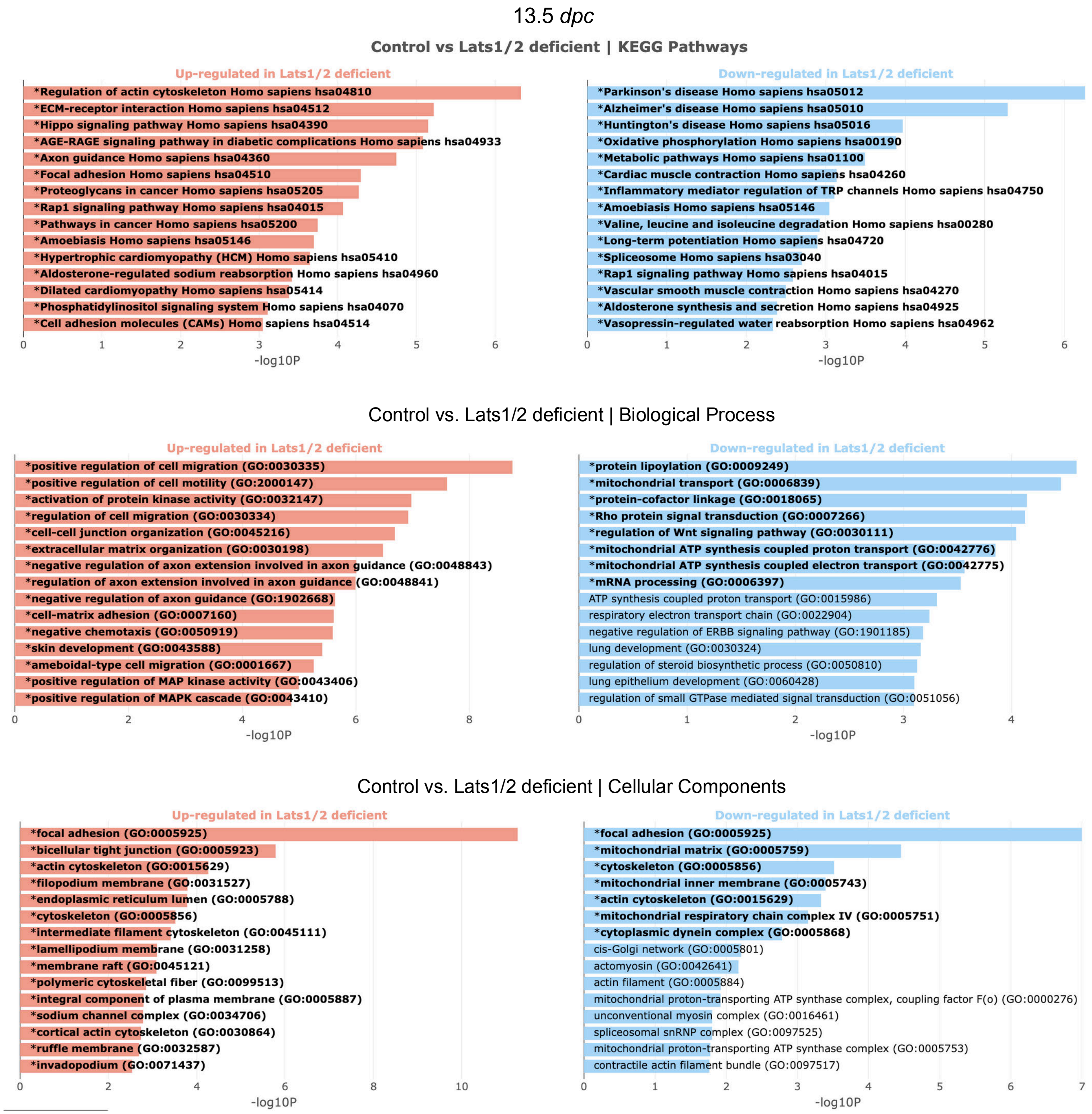

Zhang et al. Supplementary Figure 6

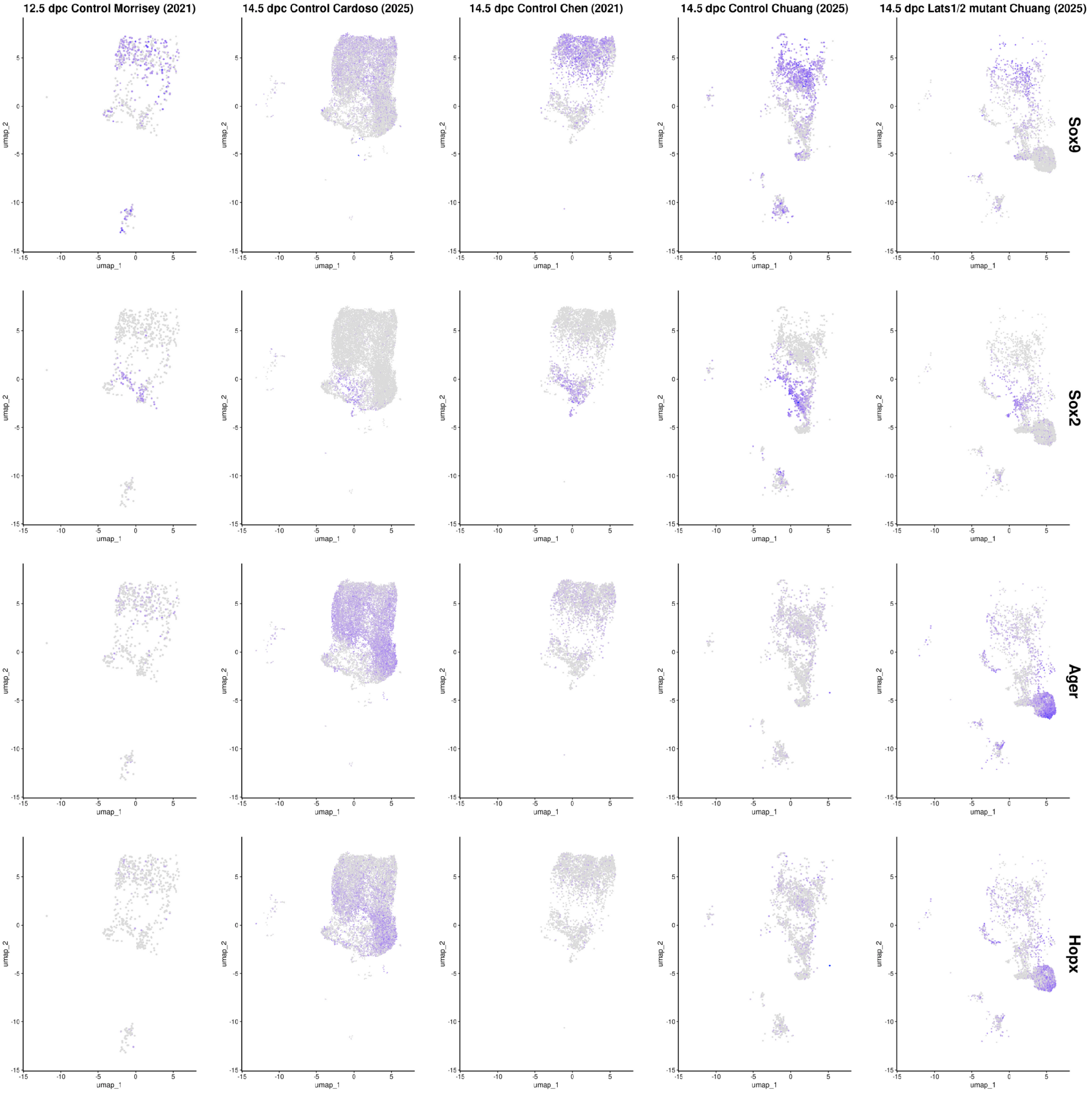

Zhang et al. Supplementary Figure 7

a

14.5 *dpc*

Control lungs (control, CT)

Lats1$^{f/f}$*; Lats2*$^{f/f}$*; Sftpc*$^{Cre/+}$ lungs (mutant, MT)

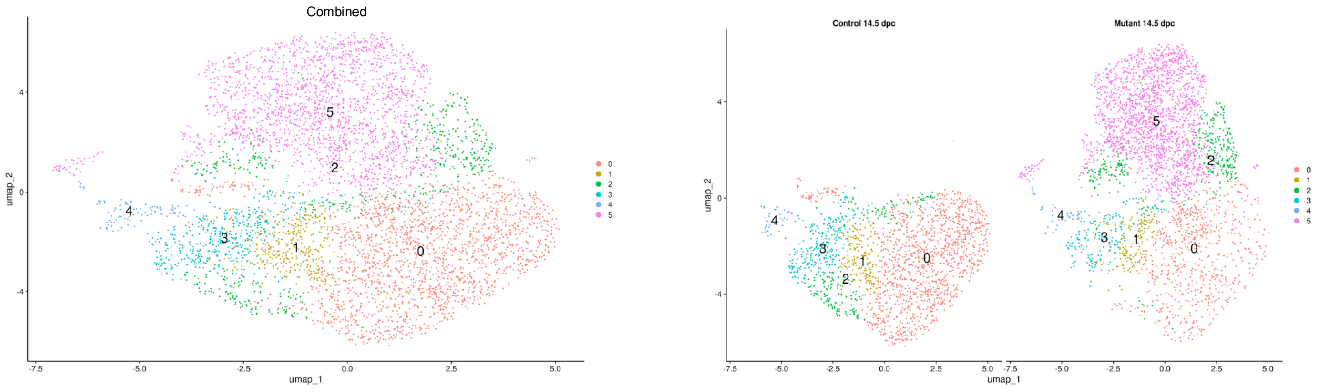

b

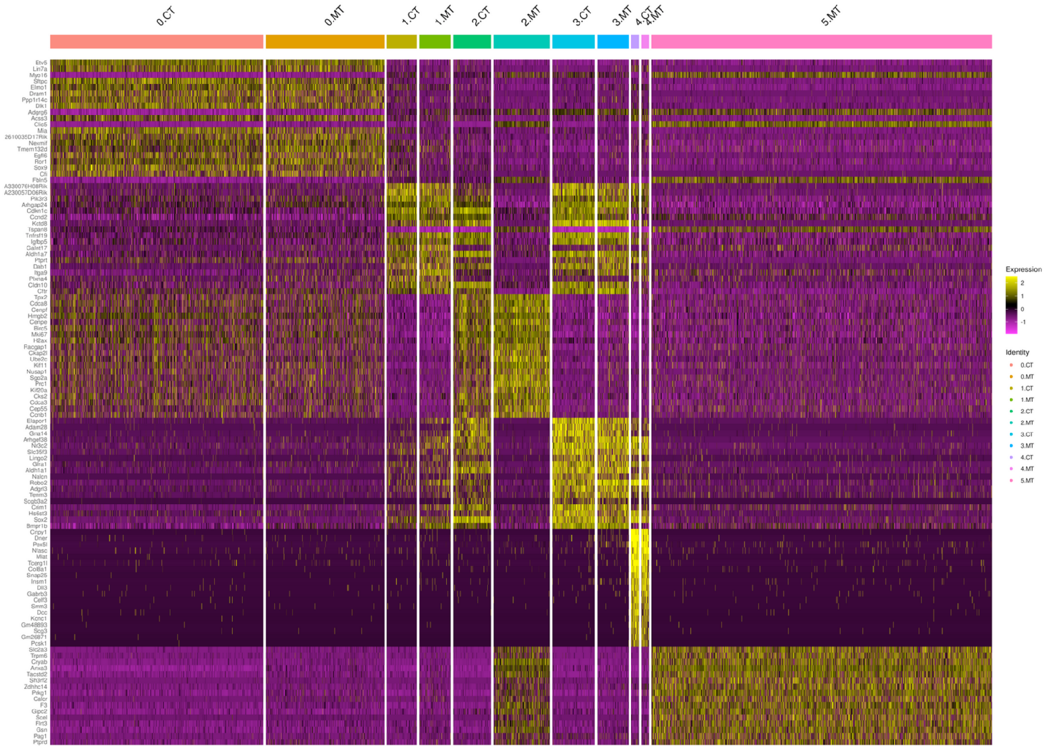

Zhang et al. Supplementary Figure 8

14.5 *dpc*

Control lungs (control, CT)

Lats1$^{f/f}$; Lats2$^{f/f}$; Sftpc$^{Cre/+}$ lungs (mutant, MT)

a

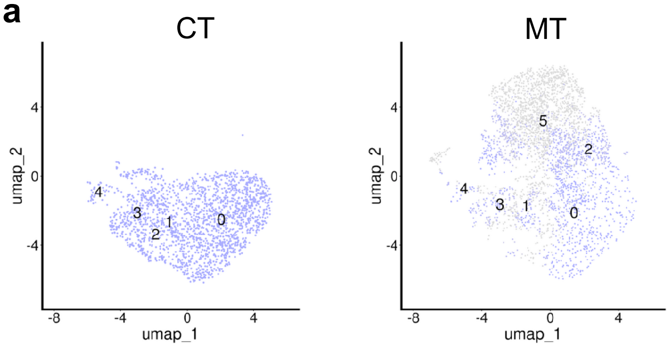

b

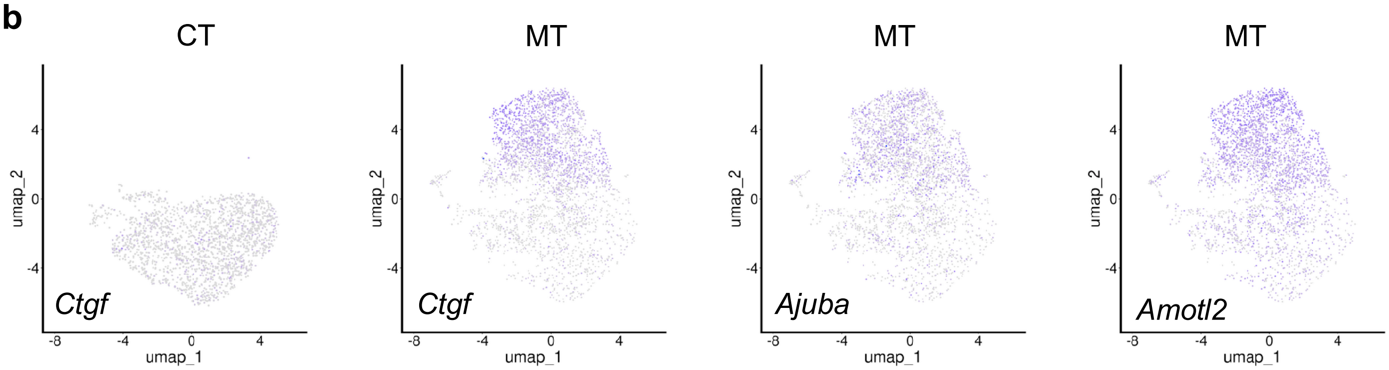

17.5 *dpc*

Control lungs (control, CT)

Lats1$^{f/f}$; Lats2$^{f/f}$; Sftpc$^{Cre/+}$ lungs (mutant, MT)

c

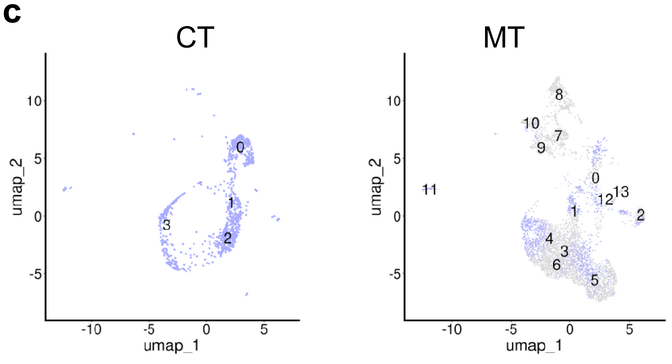

d

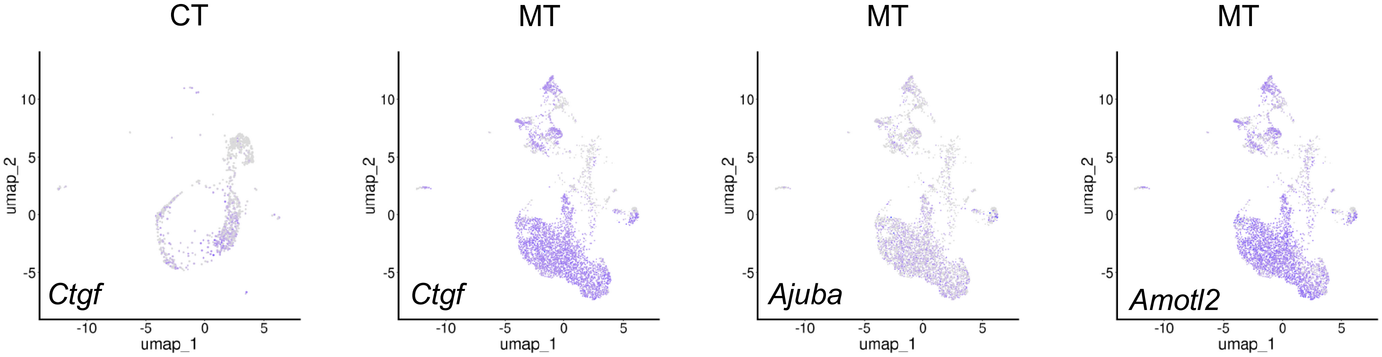

Zhang et al. Supplementary Figure 9

a

Control lungs 14.5 *dpc* Increased in expression ↑ Reduced in expression ↓

Cluster 0 —— 1	*Anxa1 Hivep2 Kdm7a Mctp2 Meis2 Mxd4 Mycl Nr3c2 Rarb Rorc Sox2 Tbx1*	↑
Cluster 0 —— 1	*Brca1 Ccdc25 Celf4 E2f7 E2f8 Etv5 Hirip3 Kif22 Lef1 Nup107 Rbpjl Scx Sp5 Tcf7*	↓
Cluster 1 —— 2	*Anxa1 Bcl6 Dach1 Hey1 Id4 Kif22 Meis2 Mycl Nr3c2 Ovol1 Six1 Sox2 Sox21 Terf1 Zbtb7c*	↑
Cluster 1 —— 2	*Creb5 Mycn Zfp521*	↓
Cluster 2 —— 3	*Npas2 Nr3c2 Trp63*	↑
Cluster 2 —— 3	*Bcl11b Brca1 Etv5 Foxm1 Hirip3 Kif22*	↓

b

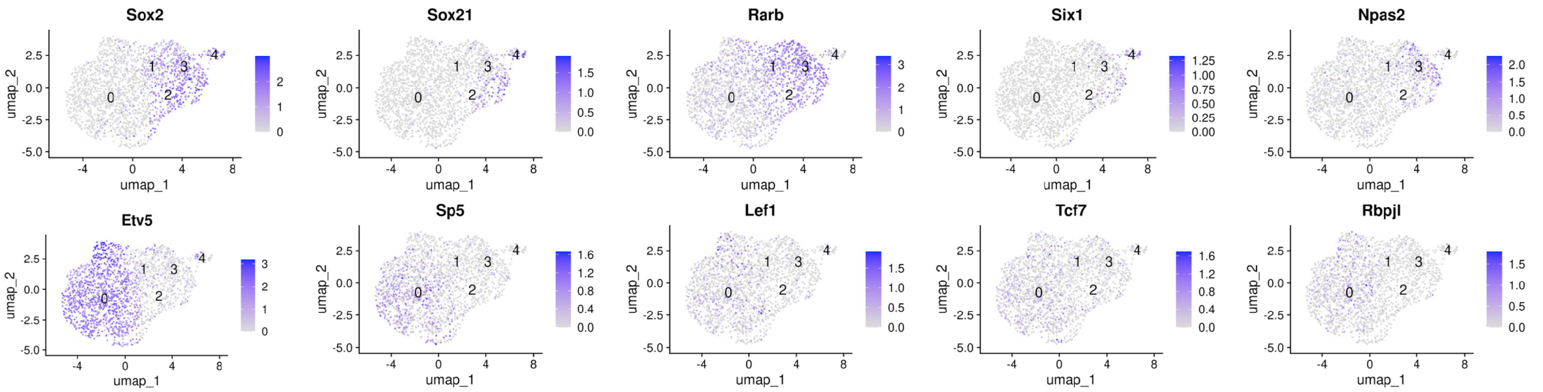

c

Lats1$^{f/f}$; *Lats2*$^{f/f}$; *Sftpc*$^{Cre/+}$ lungs 14.5 *dpc* Increased in expression ↑ Reduced in expression ↓

Cluster 0 —— 1	*Ascl1 Bcl6 Egr3 Grhl3 Hivep2 Junb Kdm7a Maf Mctp2 Meis1 Meis2 Mycl Nr3c2 Ovol1 Ppargc1a Prox1 Rarb Sox2 Tbx1*	↑
Cluster 0 —— 1	*Bcl11b Brca1 Celf4 Dnmt1 Dtl Etv5 Foxm1 Irx1 Lef1 Pold2 Rbpjl Sox9 Sp5 Z2f8 Zfp367*	↓
Cluster 0 —— 2	*Anxa1 Dmrt1 Elf4 Jdp2 Osr1 Pou6f2 Runx2 Zbtb8b Zbtb32*	↑
Cluster 0 —— 2	*Etv5 Sox9 Elf5 Lef1 Celf4 Mitf Rbpjl Hivep3 Etv4*	↓
Cluster 1 —— 3	*Anxa1 Bcl6 Elf3 Fos Id4 Mef2c Meis1 Meis2 Npas2 Ppargc1a Nr3c2 Rorb Sox21*	↑
Cluster 1 —— 3	*Creb5*	↓
Cluster 2 —— 5	*Tbx4 Zfp423 Pnox2 Frem1 Bnc2 Meis1 Zeb2 Gli2 Hivep3 Prdm6 Tbx5 Mylk Etv1*	↑
Cluster 2 —— 5	*Foxm1 Kif22 Mxd3*	↓

d

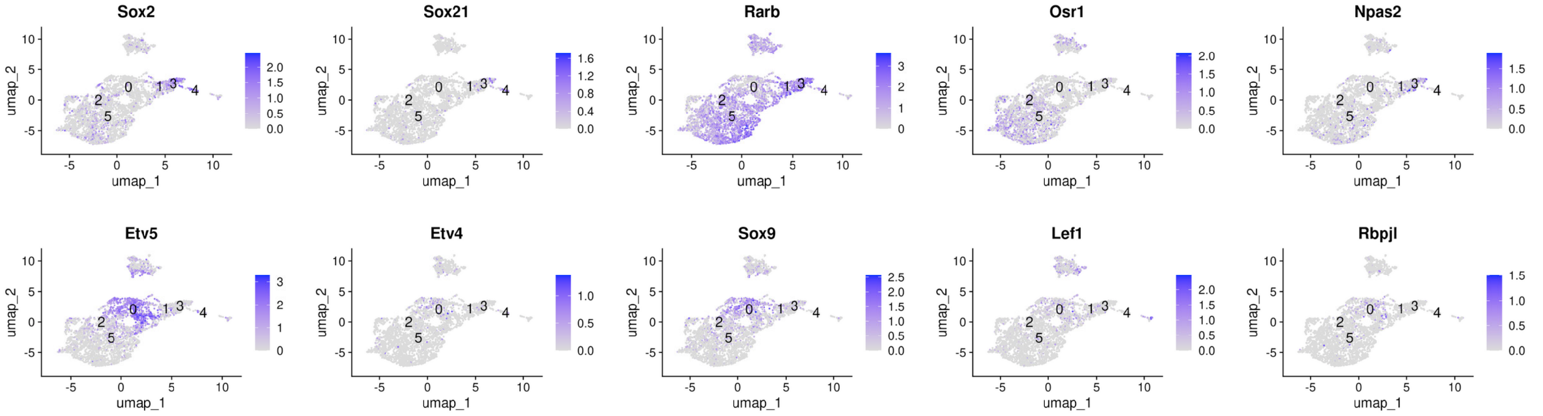

Zhang et al. Supplementary Figure 10

Control lungs 14.5 *dpc* Cluster 0 – 1

Rarb/Meis2/Mxd4/Etv5/E2f7/Sp5/Sox2

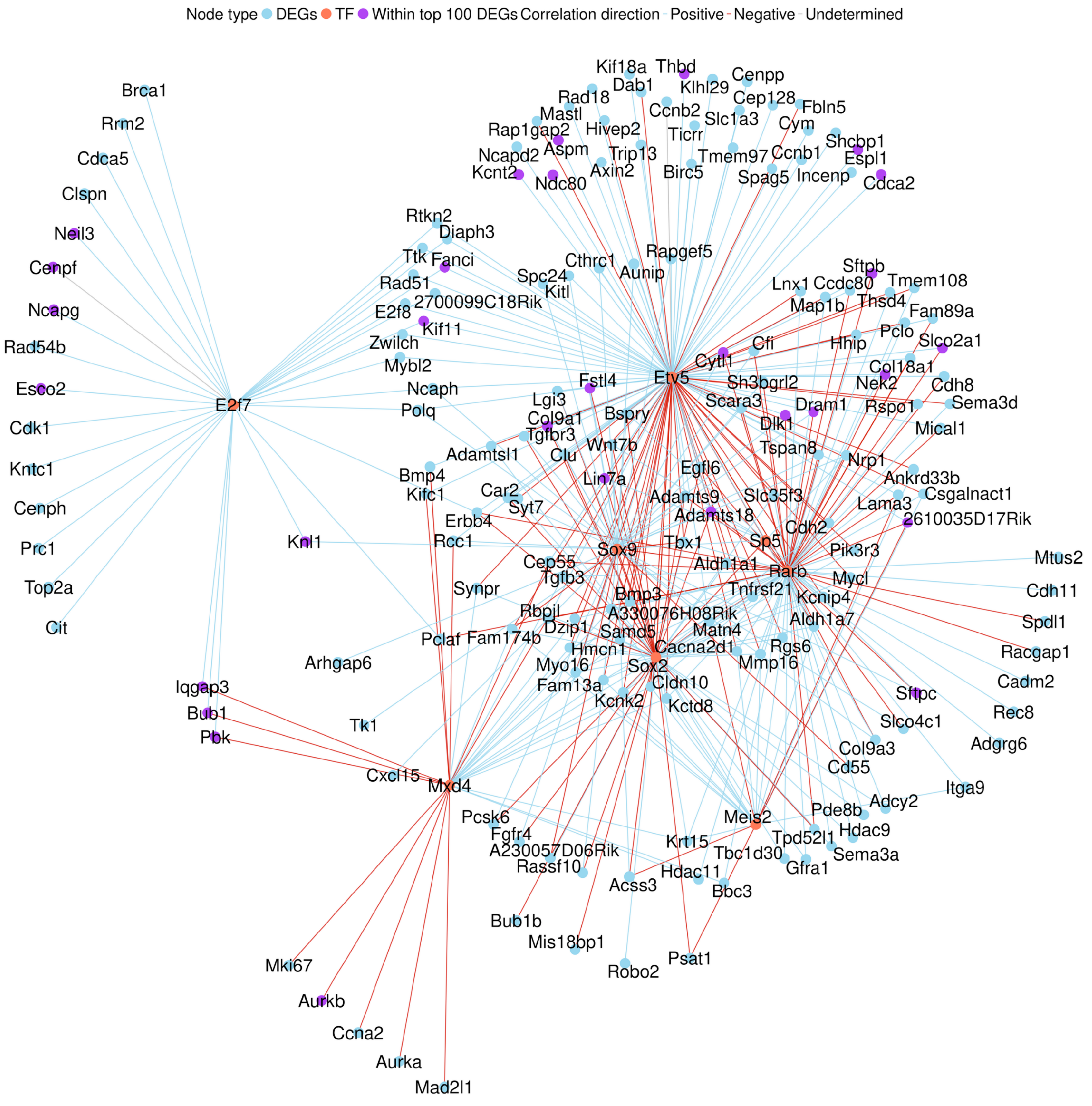

Zhang et al. Supplementary Figure 11

a

Motifs enriched in SOX9$^+$ cells of control lungs at 14.5 *dpc*

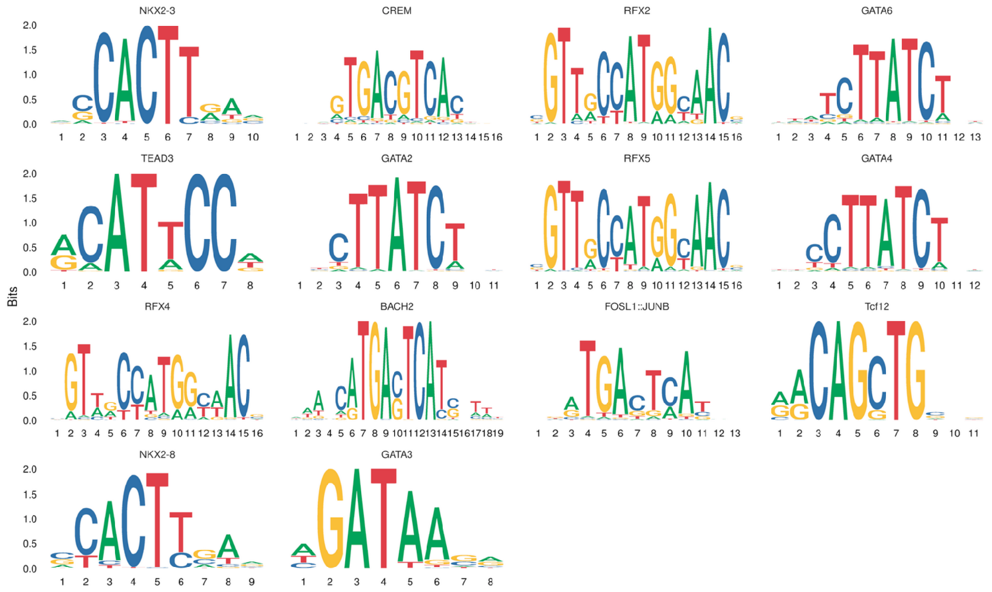

b

Motifs enriched in SOX2$^+$ cells of control lungs at 14.5 *dpc*

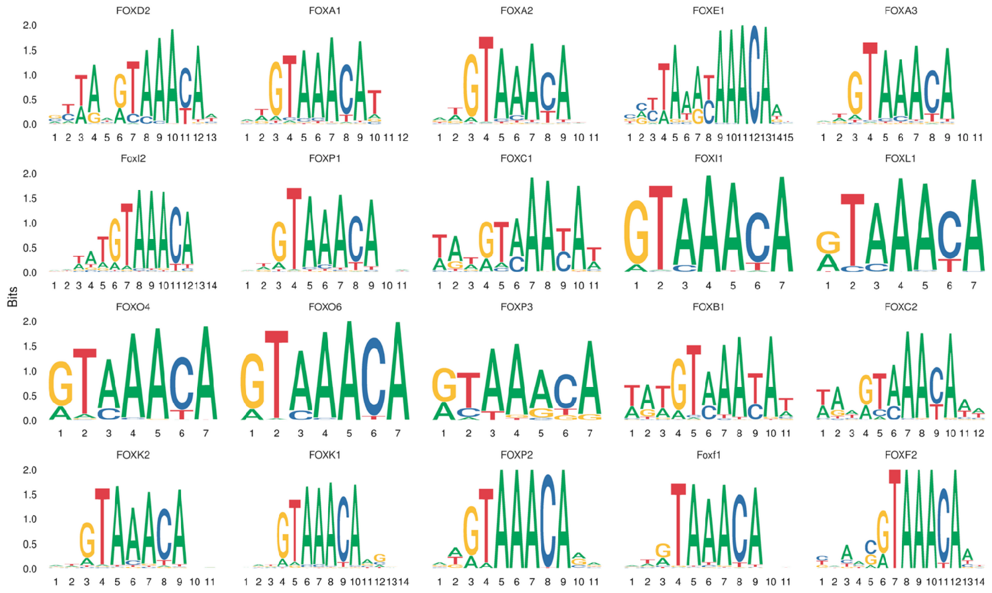

c

DEGs	Motifs	DEGs	Motifs
Rarb	TEAD	*Etv5*	TEAD
Meis2	TEAD	*E2f7*	TEAD
Mxd4	TEAD	*Sp5*	TEAD
Sox2	TEAD	*Tcf7*	TEAD
		Lef1	TEAD
		Rbpjl	TEAD

Zhang et al. Supplementary Figure 12

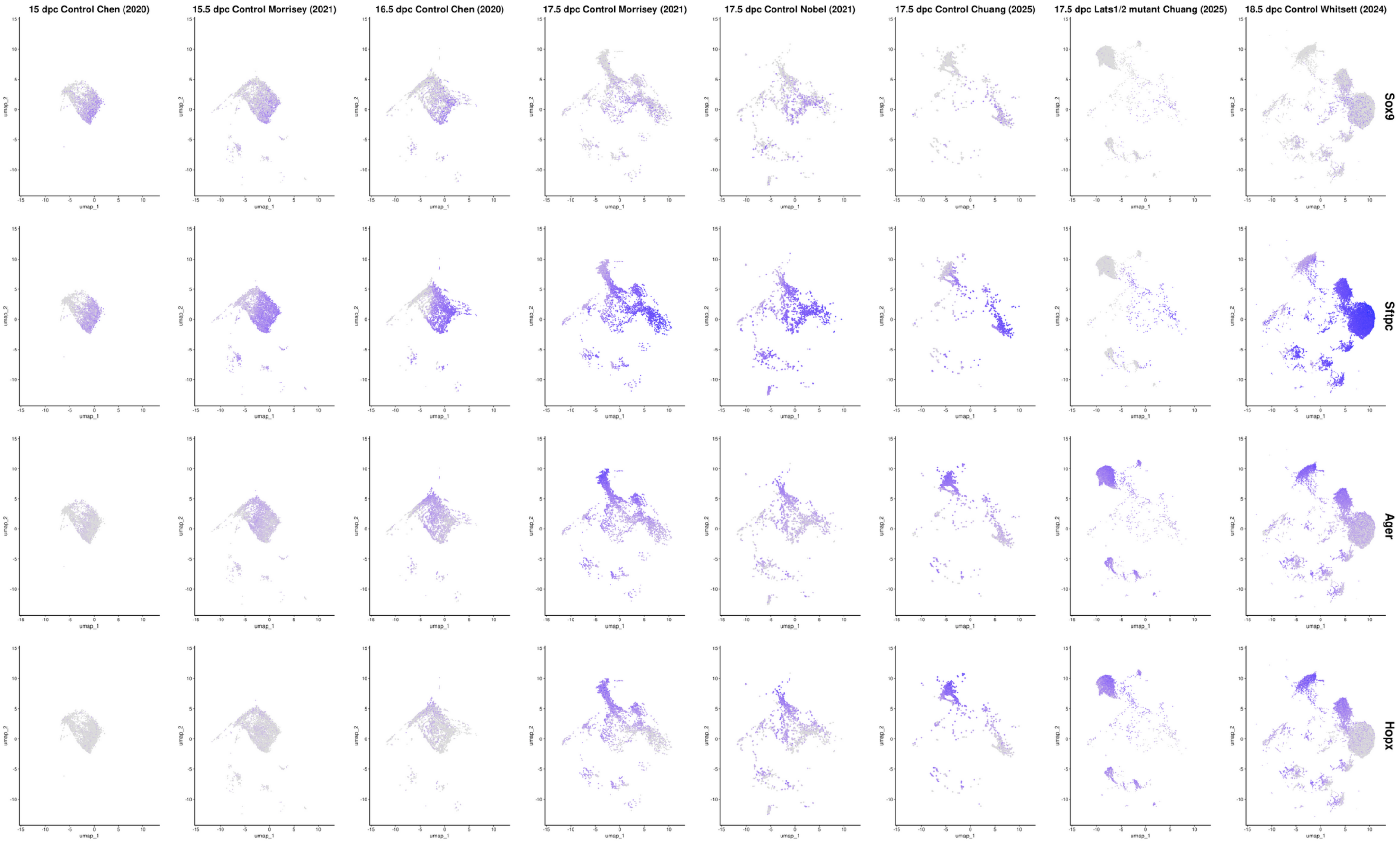

Zhang et al. Supplementary Figure 13

a 17.5 *dpc*

Control lungs

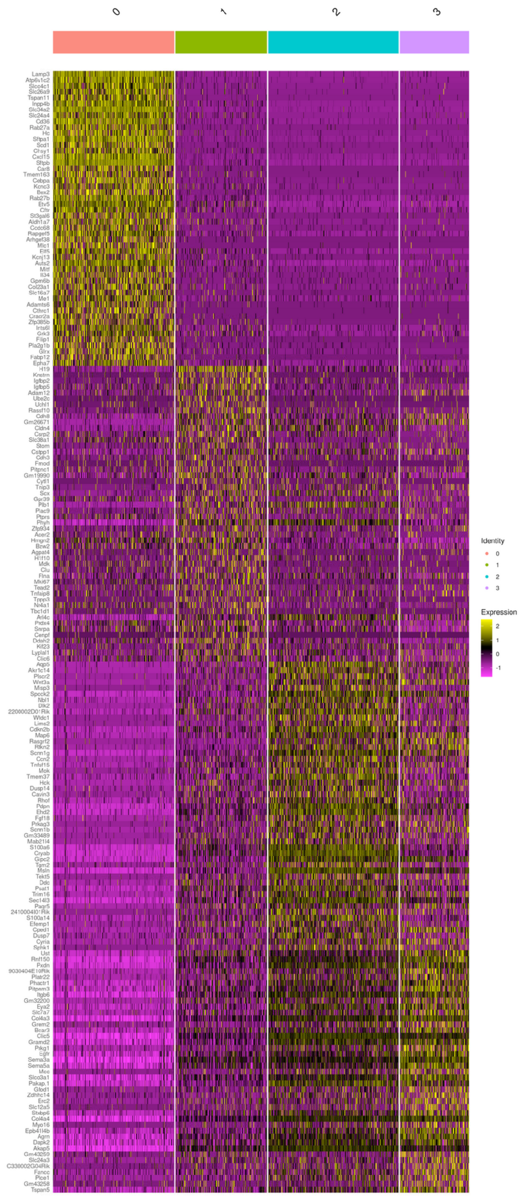

b 17.5 *dpc*

Lats1$^{f/f}$; *Lats2*$^{f/f}$; *Sftpc*$^{Cre/+}$ lungs

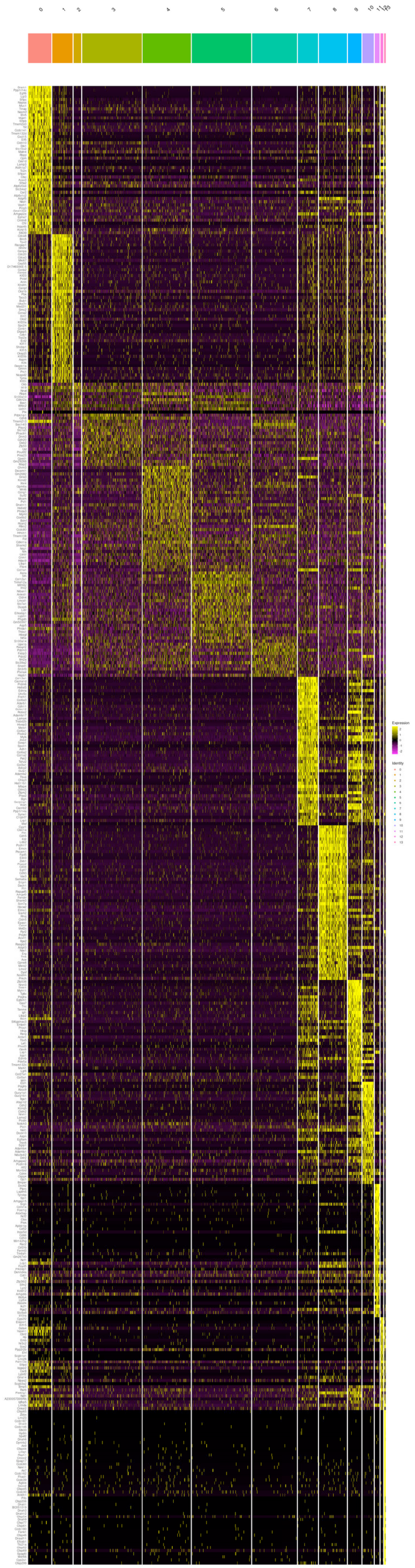

Zhang et al. Supplementary Figure 14

a

Control lungs 17.5 *dpc* Increased in expression ↑ Reduced in expression ↓

Cluster 0 —— 1	*Celf5 Cers3 E2f7 Mycn Myrf Pbx4 Rarb Scx Smad9 Sox5 TEAD4 Zbtb8b*	↑
Cluster 0 —— 1	*Arg2 Cebpa Creb3l1 Elf5 Etv5 Foxq1 Gpam Mitf Prdm1 Sp5 Tfcp2l1 Zmat4*	↓
Cluster 1 —— 2	*Prkaa2 Stat5b TEAD4 Zbtb16*	↑
Cluster 1 —— 2	*Dtl Elf5 Etv5 Hmga1 Hmgb2 Magog Nr4a1 Ran Rfc3 Rnaseh2c Sox9 Thap11 Zfp414*	↓
Cluster 2 —— 3	*2310011J03Rik Ak6 Borcs8 Csnk2b Id1 Nme1 Prdx5 Rnaseh2c Rpl35 Snapc5 Spag7 Thap11 Zbtb22 Zfp768*	↑

b

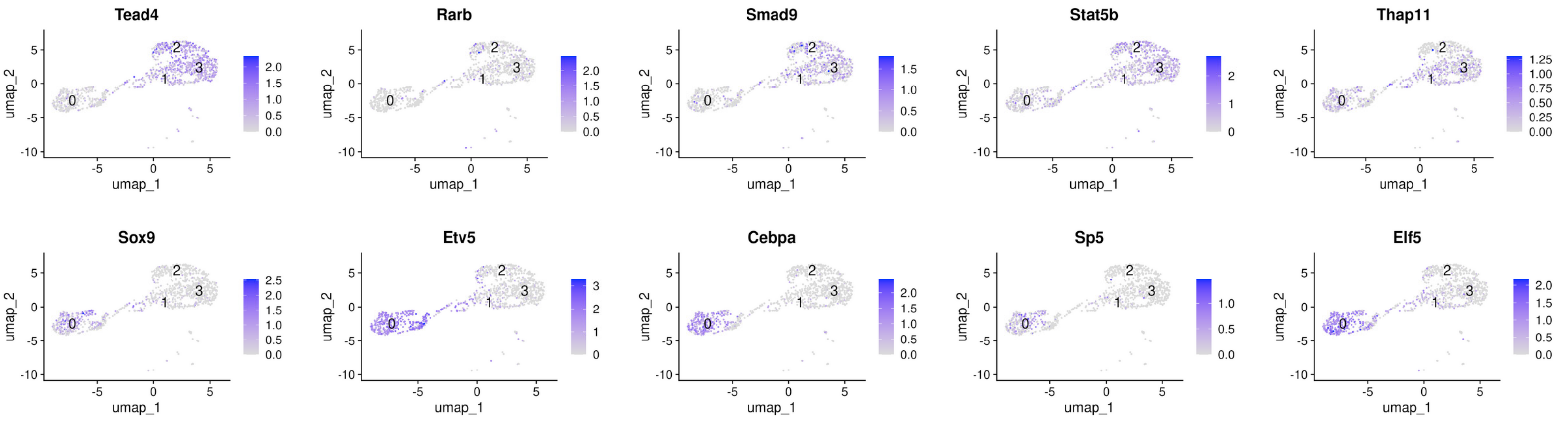

c

Lats1$^{f/f}$*; Lats2*$^{f/f}$*; Sftpc*$^{Cre/+}$ lungs 17.5 *dpc* Increased in expression ↑ Reduced in expression ↓

Cluster 0 —— 1	*Arid3b Brca1 Dab2 E2f7 E2f8 Elf4 Fhl2 Foxm1 Hirip3 Kif22 Myrf Pbx4 Plagl1 Pou6f2 Runx2 Sox11 Zbtb8b*	↑
Cluster 0 —— 1	*Arg2 Ascl2 Bcl6 Cebpa Celf4 Creb3l1 Elf5 Egr2 Epas1 Etv5 Fosb Gpam Hhex Hivep3 Id2 Irf1 Irx2 Irx3 Junb Mitf Nfe212 Nfix Npas2 Nr4a1 Ovol1 Pdrm1 Ppara Ppargc1a Rbpjl Rora Sox9 Sp5 Stat3 Thrb Zfp467 Zmat4*	↓
Cluster 1 —— 2, 3, 4, 5, 6	*Hif3a Hivep2 Kdm7a Mxd4 Prkaa2 Tef Zfp949*	↑
Cluster 1 —— 2, 3, 4, 5, 6	*Brca1 Dnmt1 Dtl E2f7 E2f8 Elf5 Foxm1 Hirip3 Id1 Kif22 Hmgb2 Mxd3 Mybl2 Timeless*	↓

d

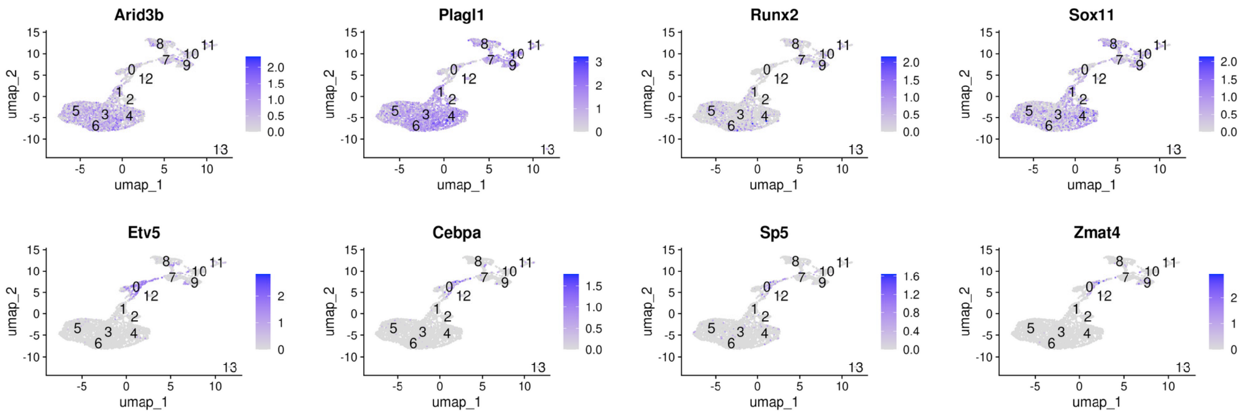

Zhang et al. Supplementary Figure 15

Control lungs 17.5 *dpc* Cluster 0 – 1

Tead4/Rarb/Cebpa/Etv5/Elf5

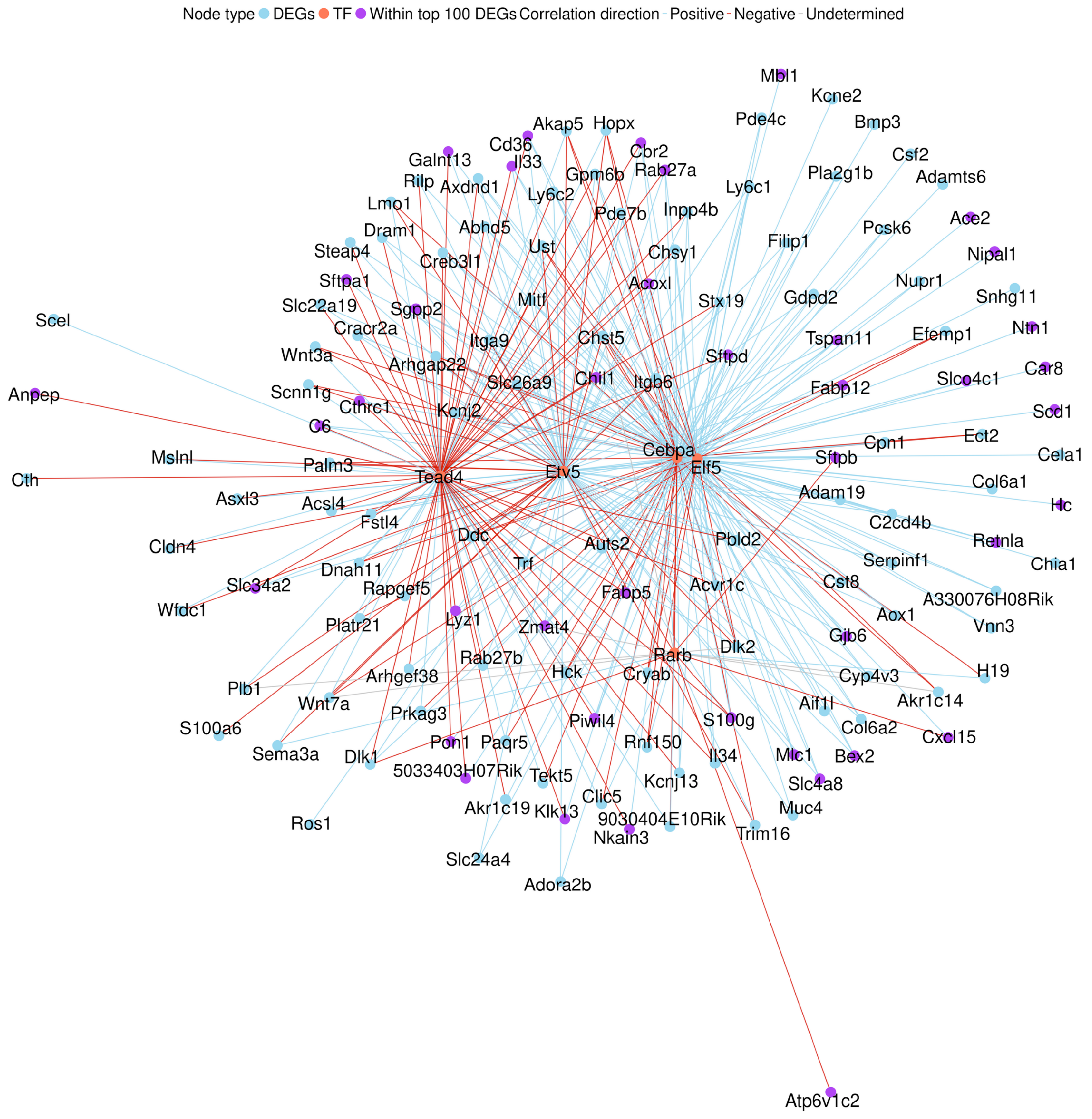

a

Motifs enriched in AT1 cells of control lungs at 17.5 *dpc*

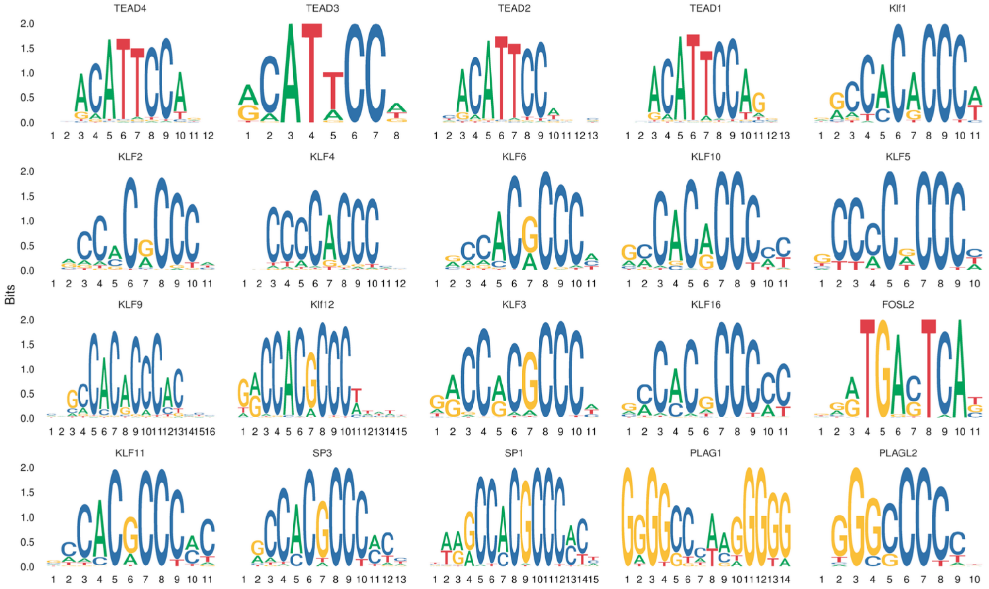

b

Motifs enriched in AT2 cells of control lungs at 17.5 *dpc*

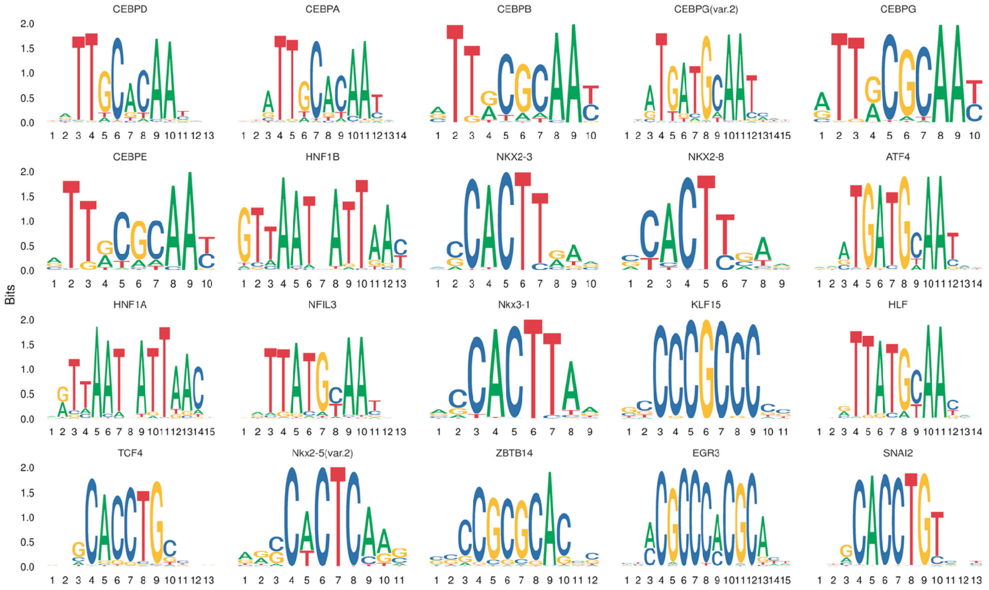

c

DEGs	Motifs	DEGs	Motifs
Arid3	TEAD	*Cebpa*	TEAD
Klf5	TEAD	*Etv5*	TEAD
Sox11	TEAD		
Runx2	TEAD		
Plagl1	TEAD		

Zhang et al. Supplementary Figure 17

a

Control mouse lungs 14.5 *dpc*

Mouse lungs cluster 0
Human lungs early tip

Mouse lungs cluster 2
Human lungs early stalk

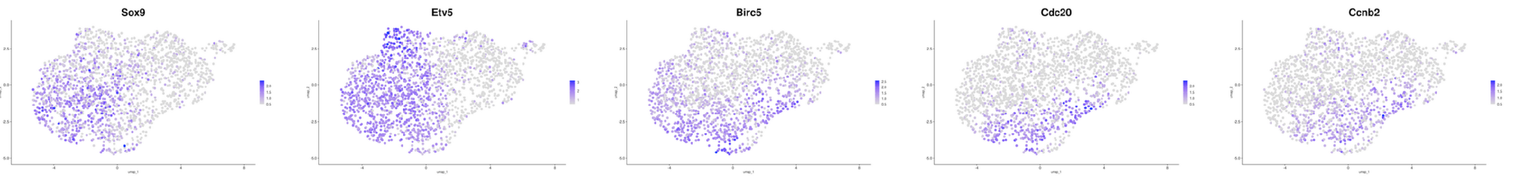

b

Lats1$^{f/f}$; *Lats2*$^{f/f}$; *Sftpc*$^{Cre/+}$ mouse lungs 14.5 *dpc*

Mouse lungs cluster 0
Human lungs early tip

Mouse lungs cluster 2
Human lungs early stalk

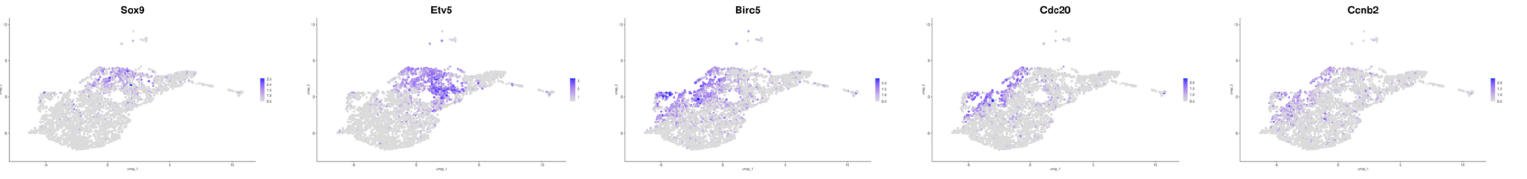

c

Control mouse lungs 17.5 *dpc*

Mouse lungs cluster 0
Human lungs late tip

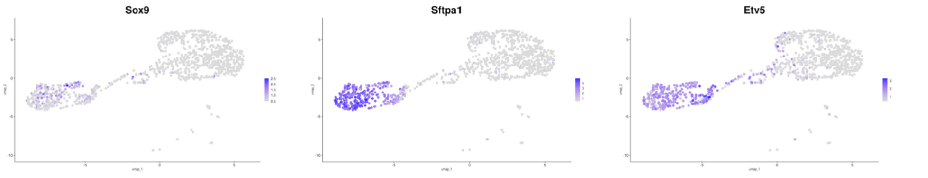

d

Lats1$^{f/f}$; *Lats2*$^{f/f}$; *Sftpc*$^{Cre/+}$ mouse lungs 17.5 *dpc*

Mouse lungs cluster 0
Human lungs late tip

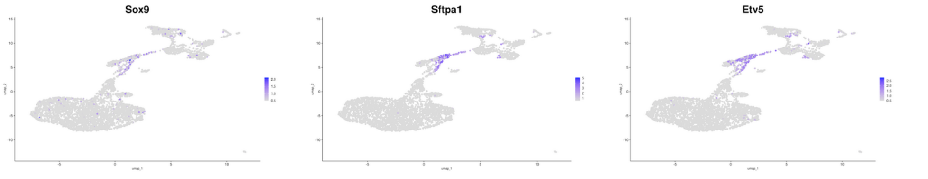

e

Control mouse lungs 14.5 *dpc*

Mouse lungs cluster 4
Human lungs neuroendocrine cells

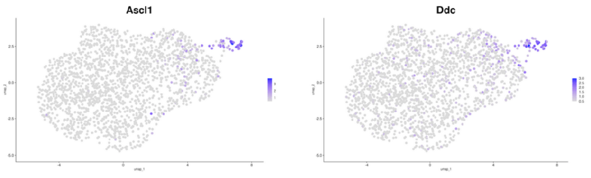

f

Lats1$^{f/f}$; *Lats2*$^{f/f}$; *Sftpc*$^{Cre/+}$ mouse lungs 14.5 *dpc*

Mouse lungs cluster 4
Human lungs neuroendocrine cells

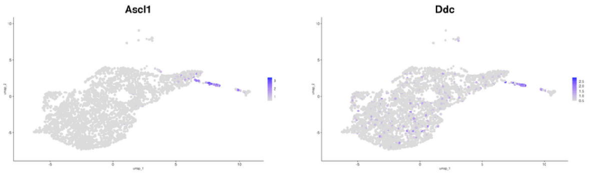

Supplement: Supplementary file 1 [file media-1.pdf]
